# THE DYNAMIC NATURE OF SUBJECTIVE AGE ACROSS THE LIFE SPAN

**DOI:** 10.1093/geroni/igac059.643

**Published:** 2022-12-20

**Authors:** David Weiss

**Affiliations:** Martin-Luther-University of Halle-Wittenberg, Halle, Sachsen-Anhalt, Germany

## Abstract

A large body of research has confirmed that from childhood to old age most individuals feel significantly younger or older than their chronological age. Up to now, however, there is no clear theoretical understanding as to why younger adults tend to feel on average older and older adults tend to feel on average younger. We adopt a motivated social-cognition perspective on subjective age and examine age-differential antecedents and correlates of subjective age across the adult life span. Results from a cross-sectional study (N = 1652, 18-84 years) and a 9-month longitudinal study (N = 814; 18-84 years) highlight the dynamic link between subjective age bias and individual (motivation and emotion) as well as social factors (social comparison, meta stereotypes). We discuss the role of reciprocal dynamics between individuals and social contexts in explaining why individuals adopt a younger or older subjective age.

